# Evaluation of prevalence, biochemical profile, and drugs associated with chronic kidney disease-mineral and bone disorder in 11 dialysis centers

**DOI:** 10.1590/2175-8239-JBN-3527

**Published:** 2018-05-07

**Authors:** Rodrigo Reis Abrita, Beatriz dos Santos Pereira, Neimar da Silva Fernandes, Renata Abrita, Rosalia Maria Nunes Henriques Huaira, Marcus Gomes Bastos, Natália Maria da Silva Fernandes

**Affiliations:** 1 Universidade Federal de Juiz de Fora, Juiz de Fora, MG, Brasil.; 2 Centro de Ensino Superior de Juiz de Fora, Passos, Juiz de Fora, MG, Brasil.

**Keywords:** Chronic renal failure, renal dialysis, bone diseases, Falência renal crônica, diálise renal, doenças ósseas

## Abstract

**Introduction:**

The diagnosis and treatment of mineral and bone disorder of chronic kidney disease (CKD-MBD) is a challenge for nephrologists and health managers. The aim of this study was to evaluate the prevalence, biochemical profile, and drugs associated with CKD-MBD.

**Methods:**

Cross-sectional study between July and November 2013, with 1134 patients on dialysis. Sociodemographic, clinical, and laboratory data were compared between groups based on levels of intact parathyroid hormone (iPTH) (< 150, 150-300, 301-600, 601-1000, and > 1001 pg/mL).

**Results:**

The mean age was 57.3 ± 14.4 years. The prevalence of iPTH < 150 pg/mL was 23.4% and iPTH > 601 pg/mL was 27.1%. The comparison between the groups showed that the level of iPTH decreased with increasing age. Diabetic patients had a higher prevalence of iPTH < 150 pg/mL (27.6%). Hyperphosphatemia (> 5.5 mg/dL) was observed in 35.8%. Calcium carbonate was used by 50.5%, sevelamer by 14.7%, 40% of patients had used some form of vitamin D and 3.5% used cinacalcet. Linear regression analysis showed a significant negative association between iPTH, age, and *diabetes mellitus* and a significant positive association between iPTH and dialysis time.

**Conclusion:**

The prevalence of patients outside the target for iPTH was 50.5%. There was a high prevalence of hyperphosphatemia (35.8%), and the minority of patients were using active vitamin D, vitamin D analogs, selective vitamin D receptor activators, and cinacalcet. These data indicate the need for better compliance with clinical guidelines and public policies on the supply of drugs associated with CKD-MBD.

## RESUMO

**Introdução:**

O diagnóstico e tratamento do distúrbio mineral ósseo-doença renal crônica (DMO-DRC) é um desafio para os nefrologistas e gestores de saúde. O objetivo deste estudo foi avaliar a prevalência, perfil bioquímico, e drogas associadas a DMO-DRC.

**Métodos:**

Estudo transversal entre julho e novembro de 2013, em 11 centros com 1134 pacientes em diálise. Dados sociodemográficos, clínicos, e laboratoriais foram comparados entre os grupos, com base em níveis do paratormônio intacto (PTHi) (< 150,151-300, 301-600,601-1000, e > 1001 pg/mL).

**Resultados:**

A idade média foi de 57,3 ± 14,4 anos, 1071 pacientes estavam em hemodiálise, e 63 em diálise peritoneal. A prevalência de PTHi < 150 pg/mL foi 23,4% e PTHi > 601 pg/mL foi de 27,1%. A comparação dos grupos mostrou que o nível de PTHi diminuiu com o aumento da idade. Pacientes diabéticos apresentaram uma maior prevalência de PTHi < 150 pg/mL (27,6%). Carbonato de cálcio foi usado por 50,5%, Sevelamer por 14,7%, 40% dos pacientes utilizaram alguma forma de vitamina D, e 3,5% utilizaram cinacalcet. A hiperfosfatemia (> 5,5mg/dL) foi observada em 35,8%. A análise de regressão linear mostrou uma associação negativa significativa entre PTHi, idade, e *diabetes mellitus* e uma associação positiva significativa com o tempo em diálise.

**Conclusão:**

A prevalência de pacientes fora do alvo para PTHi foi de 50,5%. Houve uma alta prevalência de hiperfosfatemia e um baixo uso de vitamina D ativa, análogos da vitamina D, ativadores seletivos da vitamina D, e cinacalcet. Estes dados chamam a atenção para a necessidade de uma maior conformidade com as diretrizes e políticas públicas sobre o fornecimento de medicamentos associados à DMO-DRC.

## INTRODUCTION

Brazil is the largest country in Latin America, and the only country over 100 million people with a public health system (SUS) that assures universal access to health care since 1990. According to the Brazilian Institute of Geography and Statistics (IBGE), in 2015 Brazil had a population of 203,980,840 million people, with a human development index of 0.749 and life expectancy of 74.9 years^1^. The country is undergoing a demographic change characterized by population ageing, with the elderly comprising 31 million people. The prevalence of arterial hypertension among the population over 40 years old is 40%-50%^2^. According to the International Diabetes Federation, there are nearly 11.6 million diabetic individuals in Brazil^3^. Since the Brazilian Dialysis Survey was created by the Brazilian Society of Nephrology, the number of people on dialysis in the country has increased progressively, and was estimated to be 100,397 in the 2013 survey^4^.

One of the comorbidities associated with CKD is the mineral and bone disorder (CKD-MBD), a syndrome which encompasses clinical, biochemical (calcium, phosphate, parathyroid hormone, and active vitamin D), and bone abnormalities (bone remodeling, mineralization, and bone volume), in addition to the comorbidities associated with extra-skeletal calcification and cardiovascular disturbances^5^. The broadly used term 'renal osteodystrophy' is currently used to define changes in bone histology evaluated by biopsy^5^. Based on bone remodeling, renal osteodystrophy is classified as either a high remodeling bone disease (secondary hyperparathyroidism or osteitis fibrosa) or a low remodeling bone disease (represented by osteomalacia and adynamic bone disease). In addition, a mixed bone disease can occur, which presents both high and low remodeling characteristics and is currently classified as high remodeling^5^.

Most evidence shows that adynamic bone disease characterized by low bone turnover occurs in the early stages of chronic kidney disease in a significant proportion of patients. This could be due to the initial predominance of bone turnover inhibitory conditions such as resistance to parathyroid hormone (PTH), reduced calcitriol levels, sex hormone deficiency, *diabetes mellitus*, and uremic toxins leading to suppression of various means of bone signaling. In later stages, osteitis fibrosa, a high-turnover bone disease, develops resulting mainly from secondary hyperparathyroidism^6^.

In 1998, Diaz Lopez *et al*. evaluated the epidemiology of renal osteodystrophy in 1209 bone biopsies from patients in five Ibero-American countries (Brazil, Uruguay, Argentina, Portugal, and Spain) and showed that hyperparathyroidism was more frequent in patients on dialysis in Spain and Portugal (66 and 70%, respectively), whereas mixed and low-bone remodeling lesions accounted for less than 14%. On the other hand, South American patients showed a high prevalence of mixed and low-bone remodeling lesions (37 and 51%, respectively)^7^. The study by Araújo SM *et al*. evaluated the prevalence of CKD-MBD in dialysis patients in Brazil and Uruguay and showed, through bone biopsy, an increase of prevalence of secondary hyperparathyroidism from 32.3% in 1985-1990 to 44% in 1997-2000 in 2,340 Brazilian patients^8^. According to data from the 2011 Brazilian Census of Parathyroidectomy, the prevalence of severe secondary hyperparathyroidism was 10.7%, based on levels of intact parathyroid hormone (iPTH) higher than 1000 pg/mL, indicative of parathyroidectomy^9^.

A study published in 2011 and conducted in hemodialysis centers in Argentina, Chile, Colombia, Venezuela, Mexico, and Brazil (CORES Study) involving 16,173 patients analyzed the impact of calcium, phosphate, and iPTH levels in mortality and concluded that low or high serum levels of all variables raised the risk of death^10^. Abnormalities in serum phosphorus, calcium, and parathyroid hormone have been associated with poor survival in dialysis patients. A total of 4,500 patients were randomly recruited for COSMOS study from 227 dialysis centers in 20 European countries. In summary, a non-linear relationship was found between serum CKD-MBD biochemical parameters and mortality risk. Low and high serum levels of serum phosphorus, calcium, and PTH were associated with a higher relative risk of mortality while decreases in serum phosphorus and calcium and increases in serum PTH during follow-up were associated with a significant lower risk of mortality^11^.

iPTH levels below 150 pg/mL were seen in 36% of 3,226 incident and prevalent patients treated with peritoneal dialysis in Brazil (continuous ambulatory peritoneal dialysis or automated peritoneal dialysis)^12^.

Historically, renal osteitis fibrosa and mixed uremic osteodystrophy were the most prevalent bone diseases in patients with CKD. Recently, an increase in the prevalence of adynamic bone disease has been observed, particularly in diabetic patients^6^^,^ 13.

Although abundant, the studies cited above show partial data or were based on patients followed in a reference center for CKD-MBD, thus are subject to bias. The evaluation of CKD-MBD patterns, practices, and epidemiology is fundamental for public health programs. In Brazil, there are considerable difficulties in keeping records on specific pathologies and, especially, planning strategies based on accurate information. Thus, studies assessing occurrences in the various regions of the country, characterized by different realities, are vital for better healthcare planning. Since there is a lack of an extensive evaluation of patients on hemodialysis (HD) and peritoneal dialysis PD in Brazil, the aim of this study was to evaluate the prevalence, biochemical profile, and drugs associated with CKD-MBD in these patients.

## MATERIAL AND METHODS

### STUDY DESIGN AND SETTING

We performed a cross-sectional study in 11 nephrology centers run by the Nephrology Centers Association of the State of Minas Gerais (*Associação Mineira de Centros de Nefrologia - AMICEN*), which covers 2,371,572 people^14^. The association includes 19 renal replacement therapy (RRT) centers, distributed across the East, South, Northeast, Midwest and central areas of Minas Gerais, Brazil.

The present study was performed according to the Declaration of Helsinki and approved by the Ethics and Research Committee of the Hospital of the Federal University of Juiz de Fora, Brazil (Universidade Federal de Juiz de Fora), under the following number: 21702913.9.0000.5147.

### STUDY POPULATION

The inclusion criteria were patients with CKD over 18 years old on dialysis (HD or PD) for more than 3 months, with at least one iPTH dosage in 2013 and who have signed the informed consent form. The exclusion criteria were patients less than 18 years old and who were missing laboratorial iPTH dosage within the period of study.

### STUDY PROCEDURES AND MEASUREMENTS

Data were based on medical records and on the laboratorial evaluation obtained in July and November 2013. For this purpose, the NEFRODATA^®^ software (medical registry) was used. The following socio-demographic variables were analyzed: age, sex, race, income, and level of education (according to the IBGE). The following clinical variables were analyzed: CKD etiology, associated comorbidities, and dialysate calcium concentration. Laboratorial variables included hemoglobin (g/dL), urea (UV enzymatic method - mg/dL), creatinine (kinetic Jaffe method - mg/dL), albumin (bromocresol green colorimetric method - g/dL), iron (ferrozine colorimetric method - µg/dL), total serum calcium (Arsenazo III method - mg/dL), serum phosphate (phosphomolybdate UV method - mg/dL), alkaline phosphatase (colorimetric method - modified from Bowers and McComb - U/L), iPTH (chemiluminescence method - pg/mL) and aluminum (atomic absorption spectroscopy method with Zeeman correction - µg/L).

The following drugs used in CKD-MBD therapy were also evaluated: phosphate binders (calcium carbonate, calcium acetate, and sevelamer), active vitamin D (calcitriol oral and venous), vitamin D analogues (alfacalcidol), selective vitamin D receptor activators (paricalcitol), and calcimimetics (cinacalcet). The number of parathyroidectomies conducted in 2013 was also recorded.

### STATISTICAL ANALYSIS

A descriptive analysis was initially carried out and the data were reported by the mean±standard deviation, median (interquartile range) or percentage, depending on the variable. The population was divided based on iPTH levels (< 1 50; 151-300; 301-600; 601-1000 > 1000 pg/mL) according to Douthat *et al*. who published a study based on 25 dialysis centers in Argentina^15^. The socio-demographic, clinical, and laboratorial data were compared between groups through ANOVA, chi-square or Mann-Whitney tests. The comparison between dialysis modality vs laboratorial variables and *diabetes mellitus* (yes and no) vs laboratorial variables by Student's t-test or chi-square. Linear regression analysis was used to determine which variables influenced iPTH levels. Pearson's or Spearman's correlations were performed between the levels of iPTH and clinical and laboratory variables. The criteria for inclusion in multivariate regression was statistical and/or clinical significance. iPTH dosage was used as the dependent variable and the following predictor variables were used in model 1: age, *diabetes mellitus*, duration of therapy, and therapy modality. A confidence interval of 95% with *p* < 0.05 was used and the software was IBM's SSPS 15.0.

## RESULTS

Of the 19 AMICEN dialysis centers invited to participate in the study, 11 provided data on their patients. Although 1219 patients were initially considered eligible, 85 were later excluded from the study for one of the following reasons: less than 18 years old, on dialysis for less than 3 months or missing laboratorial iPTH dosage within the study period. Out of the 1134 patients evaluated, the mean age was 57.3 ± 14.4 years, varying from 19-96, 55.6% were males, 67% had elementary school education, and the monthly median income was US$ 274. The socio-demographic characteristics of the patients according to iPTH levels are summarized in Table 1.

**Table 1:** Socio-demographic and clinic characteristics of the total population (n = 1134) and divided by PTHi level group

Variables	Population n = 1134	PTHi (pg/mL)					*p*
		< 150 (n = 265 - 23.4%)	150-300 (n = 264 - 23.3%)	300-600 (n = 298 - 26.2%)	600-1000 (n = 173 - 15.3%)	>1000 (n = 134 - 11.8%)	
**Age (years)** (Mean ± standard deviation)	60.4 ±14.9^a-b,c,d,e^	58.3 ±14.3^b-a,c,d,e^	57.6 ±14.1^c-a,b,d,e^	55.9 ±13.6^d-a,b,c,e^	51.4 ±13.2^e-a,b,c,d^	60.4 ±14.9^a-b,c,d,e^	< 0.001
**Gender (%)**							0.64
Male	55.6	22.9	23.5	27.6	14.1	11.9	
Female	44.4	24.1	24.5	24.5	16.7	11.7	
**Race (%)**							< 0.001
White	48.9	23.4^a-b,c,d,e^	28.4^b-a,c,d,e^	25.0^c-a,b,d,e^	13.2^d-a,b,c,e^	10.0^e-a,b,c,d^	
Black	24.9	18.9	15.7	28.5	19.9	17.1	
Brown	26.2	27.8	20.7	26.4	14.9	10.2	
**Education (%)**							0.26
Illiterate	8.1	18.1	30.6	27.8	19.4	4.2	
Elementary School	67.0	20	22.4	30.5	16.0	11.1	
High School	20.0	21.5	26.0	24.9	13.0	14.7	
College	4.9	30.2	18.6	25.6	11.0	14.0	
**CKD etiology (%)**							< 0.001
Hyp. Nephropathy^#^	40.9	23.9^a-b,d,e^	20.8^b-a,c,d,e^	24.9^c-s,d,e^	18.6^d-a,b,c,e^	11.8^e-a,b,c,d^	
DRD^#^	24.3	27.6	26.4	32.9	10.6	2.4	
GN^#^	13.7	16.5	20.9	23.7	19.4	19.4	
APKD^#^	3.5	25.7	22.9	31.4	8.6	11.4	
Unknown	4.6	12.8	19.1	31.9	14.9	21.3	
Others	12.9	20	29.2	26.2	13.8	10.8	
**Comorbidities (%)**							
Arterial Hypertension	80.4	22.8	22.1	26.5^c-d,e^	15.3	13.3	0.01
Diabetes Mellitus	32.9	27.8	26.4	29.5^c-d,e^	11.6	4.7	<0.001
CVD*	11.8	23.1	22.2	29.9	15.4	9.4	0.84
Stroke	3.7	24.3	21.6	32.4	13.5	8.1	0.88
PVD*	5.7	22.8	28.1	29.8	15.8	3.5	0.24
COPD*	3.1	22.6	22.6	16.1	29.0	9.7	0.32
SLE*	1.5	26.7	20.0	13.3	33.3	6.7	0.39
Hepatic Cirrhosis	0.3	0	66.7	0	0	33.3	0.17
Others	34.3	23.0	26.8	25.9	14.5	9.8	0.50
**Dialysis Modality (%)**							
Hemodialysis	94.4	22.8	23.7	26.4	15.5	11.6	0.17
Peritoneal Dialysis	5.6	33.3	15.9	23.8	11.1	15.9	
**Therapy time (months)**							<0.001
(Mean ± standard deviation)	61.9 ±54.0	53.7±52.0^a-b,d,e^	55.2 ±48^b-a,c,d,e^	53.5±49.5^c-b,d,e^	80.7±59.1^d-a,b,c,e^	85.9±58.5^e-a,b,c,d^	
**Dialysate Calcium Concentration (%)**							<0.001
3.5%	16.7	35.1	23.9	17.6	13.8	9.6	
3.0%	37.4	27.0	22.0	22.9	16.1	12.1	
2.5%	45.8	16.0	24.3	31.9	15.3	12.5	

# Hyp. Nephropathy- Hypertensive Nephrophaty; DKD: diabetic kidney disease; GN: glomerulonephritis; APKD: adult polycystic kidney disease;

* CVD: cardiovascular

The main etiology of CKD was hypertensive nephropathy (40.9%), followed by diabetic renal disease (24.3%). The majority of the patients presented with hypertension (80.4%), 32.9% were diabetic, and 11.8% had cardiovascular disease. Regarding dialysis treatment, 1071 patients were on HD (94.4%) and 63 were on PD (5.6%). The median duration of dialysis was 43 months (21-88), varying from 4 and 274 months of therapy (Table 1). PD patients were analyzed separately and no difference was found compared to the analysis in association with HD patients.

By comparing the different iPTH groups, it was observed that iPTH level decreases with increasing age (60.4 ± 14.9 *vs*. 51.4 ± 13.2, *p* < 0.0001). Patients who had diabetic kidney disease had a higher prevalence of iPTH lower than 150 pg/mL (27.6%) and a lower prevalence of iPTH greater than 1000 pg/mL (2.4%). Patients with *diabetes mellitus*, either as the cause of CKD or as comorbidity, had a higher prevalence of iPTH < 150 pg/mL (27.8%) and lower prevalence of iPTH > 1000 pg/mL (4.7%).

A higher percentage of patients with iPTH < 150 pg/mL was found in PD compared with HD (33.3% *vs*. 22.8% with *p* = 0.19); however, the difference was statistically significant, probably due to the low number of patients on PD. A longer duration of therapy was associated with a higher iPTH serum level (53.7 *vs*. 85.9 months with *p* < 0.001, Table 1).

The majority of the patients used calcium carbonate as a phosphate binder (50.5%), and 14.7% used non-calcium-based binders (Sevelamer). Over 40% of the patients used active vitamin D (Calcitriol), vitamin D analogues (Alfacalcidol) or selective vitamin D receptor activators (Paricalcitol) (Table 2).

**Table 2:** Drug theraphy in patients with CKD-MBD stratified by their levels of PTHi

Drugs	Population n = 1134	PTHi (pg/ml)					*p*
		< 150	150-300	300-600	600-1000	> 1000	
		(n = 265 - 23.4%)	(n = 264 - 23.3%)	(n = 298 - 26.2%)	(n = 173 - 15.3%)	(n = 134 - 11.8%)	
Calcium Carbonate	50.5	27.0^a-b,c,d,e^	22.4^b-a,c,d,e^	26.3^c-a,b,d,e^	15.3^d-a,b,c,e^	9.1^e-a,b,c,d^	0.007
Calcium Acetate	5.1	17.3	34.6	19.2	15.4	13.5	0.28
Sevelamer*	14.7	14.0^a-b,c,d,e^	23.8^b-a,c,d,e^	20.1^c-a,b,e^	20.7^d-a,b,e^	21.3^e-a,b,c,d^	< 0.001
Alfacalcidol**	18.7	13.3^a-b,c,d,e^	25.6^b-a,c,d,e^	30.3^c-a,b,d,e^	18.5^d-a,b,c,e^	12.3^e-a,b,c,d^	0.002
Calcitriol (oral)***	8.0	10.1^a-b,c,d^	18.0^b-a,c,d,e^	41.6^c-a,b,d,e^	19.1^d-a,b,c,e^	11.2^e-b,c,d^	0.001
Calcitriol (venous)***	13.5	7.3^a-b,c,d,e^	9.9^b-a,c,d,e^	27.2^c-a,b,d,e^	32.5^d-a,b,c,e^	23.2^e-a,b,c,d^	< 0.001
Paricalcitol****	0.2	0	50.0	0	0	50.0	0.35
Cinacalcet*****	3.5	2.5^a-b,c,d,e^	7.5^b-a,c,e^	17.5^c-a,b,d,e^	7.5^d-b,c,e^	65.0^e-a,b,c,d^	< 0.001
Erythropoietin	83.1	22.9^a-c,d,e^	22.5^b-c,d,e^	25.4^c-a,b,d,e^	16.4^d-a,b,c,e^	12.8^e-a,b,c,d^	0.01

* non-calcium-based binders;

** vitamin D analogues;

*** vitamin D oral and venous;

**** vitamin D selective receptor activators;

***** calcimimetic drugs.

Table 2 also shows that 44.3% of the patients with iPTH < 150 pg/mL were using calcium-based phosphate binders, of whom 14% used non-calcium-based binders. In relation to vitamin D, the largest percentage of patients with iPTH > 600 pg/mL used intravenous calcitriol (55.7%). Patients with iPTH < 150pg/mL used some form of vitamin D (30.7%). Calcimimetics were used by 3.5% of the patients and 72.5% had iPTH > 600 pg/mL.

Table 3 shows the laboratorial variables for the different iPTH levels. Urea and serum creatinine levels were lower in patients with iPTH < 150 pg/mL (p < 0.001), without differences in Kt/V (*p* = 0.34) or in serum albumin (*p* = 0.74). The levels of serum calcium, phosphate, and alkaline phosphatase increased with the increasing serum level of iPTH (*p* < 0.001 for all variables).

**Table 3:** Laboratorial variables and Kt/V expressed as mean ± SD in the patients studied who were stratified by their levels of PTHi

Laboratorial variables e Kt/V	Populationn = 1134	PTHi (pg/ml)					*p*
		< 150 (n = 265 - 23.4%)	150-300 (n = 264 - 23.3%)	300-600 (n = 298 - 26.2%)	600-1000 n = 173 - 15.3%)	> 1000 (n = 134 - 11.8%)	
Urea (mg/dL)	118.5 ± 37.8	110.7 ± 37.5^a-b,c,d,e^	115.9 ± 35.3^b-a,c,d,e^	119.8 ± 38.5^c-a,b,d,e^	126.5 ± 38.9^d-a,b,c^	125.3 ± 36.9^e-a,b,c,^	<0.001
Creatinine (mg/dL)	9.1 ± 3.5	8.0 ± 3.1^a-b,c,d,e^	8.6 ± 3.3^b-a,c,d,e^	9.7 ± 4.2^c-a,b,d,e^	10.0 ± 2.9^d-a,b,c^	10.2 ± 3.1^e-a,b,c^	<0.001
Calcium (mg/dL)	9.3 ± 0.9	9.5 ± 1.0^a,c^	9.3 ± 0.9	9.1 ± 0.9^c,a,e^	9.4 ± 0.9	9.5 ± 0.8^e,c^	<0.001
Phosphorus (mg/dL)	5.1 ± 1.6	4.8 ± 1.4^a-c,d,e^	4.8 ± 1.4^b-c,d,e^	5.2 ± 1.7^c-a,b,d,e^	5.5 ± 1.6^d-a,b,c,e^	5.8 ± 1.6^e-a,b,c,d^	<0.001
Hemoglobin (g/dL)	11.0 ± 2.3	10.9 ± 1.9	11.0±1.9	11.2± 3.3	11.2 ± 1.7	10.6 ± 1.7	0.14
Albumin (g/dL)	3.8 ± 0.4	3.86 ± 0.5	3.88 ± 0.4	3.88 ± 0.4	3.83 ± 0.4	3.83 ± 0.4	0.74
Iron (µg/dL)	74.1 ± 39.0	70.20 ± 34.7	75.21 ± 38.5	76.03 ± 35.1	74.57 ± 38.9	75.09 ± 53.7	0.45
Ferritin (ng/mL)	510.0 ± 402.9	511.4 ± 394.3	514.8 ± 394.1	508.0 ± 377.5	491.8 ± 443.7	526.3 ± 440.4	0.96
Alk. Phos. (U/L)#	199.7 ± 243.1	121.9 ± 101.4^a-b,c,d,e^	150.8 ± 150.3^b-a,c,d,e^	173.3 ± 143.3^c-a,b,d,e^	228.7 ±245.0^d-a,b,c,e^	470.4 ± 470.1^e-a,b,c,d^	<0.001
Aluminum (ug/L)	10.6 ± 7.0	11.2 ± 7.1	10.2 ± 6.2	10.5 ± 7.7	11.4 ± 7.4	9.1 ± 5.6	0.05
Kt/V	1.49 ±0.48	1.45 ± 0.51	1.54 ± 0.52	1.49 ± 0.48	1.49 ± 0.40	1.51 ± 0.37	0.34

# Alk. Phos. - Alkaline Phosphatase.

The median iPTH in the studied population was 327.7 pg/mL (P25:161.2 pg/mL - P75: 647 pg/mL), varying from 1.3-3264 pg/mL. The prevalence of patients with iPTH > 600 pg/mL was 27.1% and with PTH < 150 pg/mL was 23.4%. Considering KDIGO targets for iPTH, 49.5% of the patients were in the 150-600 pg/mL range. Mean serum calcium was 9.3 ± 0.9 mg/dL and serum phosphate 5.1 ± 1.6 mg/dL. Hyperphosphatemia (phosphate concentration above 5.5 mg/dL) occurred in 35.8% of the studied population and hypophosphatemia (phosphate concentration below 3.5 mg/dL) in 13.5%. Analysis of serum calcium showed that 39.3 % of the patients had a concentration above 9.5 mg/dL (hypercalcemia) while in 12.6 % it was lower than 8.4 mg/dL (hypocalcemia).

Concerning therapy modality, 11.6% of patients on HD and 15.9% on PD had a iPTH above 1000 pg/mL, with 33.3% of the patients on PD presenting a iPTH < 150 pg/mL (Table 1). Hyperphosphatemia had a high prevalence both in patients on HD (35.5%), and on PD (41.4%).

A significant positive correlation was observed between calcium concentration in the dialysate and iPTH levels (r = 0.08 *p* = 0.006), alkaline phosphatase (r = 0.490 *p* < 0.0001), and phosphorus (r = 0.234 *p* < 0.0001).

Linear regression analysis demonstrated a significant negative association in model 1 between iPTH level and age and mellitus diabetes. The duration of therapy showed a significant positive association with the iPTH serum levels (Table 4). During 2013, there were no reports of parathyroidectomy in any of the patients of the study.

**Table 4:** Linear Regression using the PTHi level as dependent variable

Model 1	Beta	Sig	Confidence interval 95 % for B	
			Inferior limit	Superior limit
Age	-0.161	0.000	-7.235	-3.410
*Diabetes Mellitus* (yes)	-0.104	0.001	-165.884	-45.411
Time of therapy	0.188	0.000	1.130	2.170
Dialysis Modality (hemodialysis)	0.055	0.060	-5.294	245.816

## DISCUSSION

In this study the prevalence of patients with iPTH < 150 pg/mL was 23.4% and with iPTH > 600 pg/mL was 27.1%. The phosphate binders used by the majority of patients in relation to the iPTH level were calcium-based, most had vitamin D by oral route, and a small percentage used calcimimetics and selective vitamin D receptor activators.

Mean age and gender, and the main etiologies in our study are similar to those of the population on dialysis in Brazil^4^ and in Argentina, as shown in a study published in 2013, which reported on 1210 patients from 25 dialysis centers, with a mean age of 55.3 ± 17.6 years and 60.8% of males^15^.

The sample used in this study represents around 1% of the Brazilian population on dialysis in 2013^4^. Regarding the modality of dialysis, our study had less patients on PD compared to the 2013 Brazilian Chronic Dialysis Survey (5.6% *vs*. 9.2%)^4^.

A study conducted with 5008 patients on dialysis in Hungary demonstrated an inverse relationship between age and iPTH levels, regardless of *diabetes mellitus* 16. Our results corroborate this conclusion, showing that age is inversely associated with iPTH level. This is probably due to a higher prevalence of low remodeling bone disorders^13^^,^ 17.

Race in the Brazilian population is a complex and controversial subject, since Brazil has the largest miscegenation in the world^18^. The lowest levels of iPTH in white and brown individuals and the highest levels in black individuals as observed in our study do not allow inferences about the general Brazilian population. As demonstrated by Dos Reis *et al*. in bone biopsies of individuals without CKD, histomorphometric analysis results showed no association with gender and race in Brazil^19^. A study performed in the United States with 2056 patients reported that black patients in pre-dialytic and dialytic stages had lower serum levels of 25 hydroxyvitamin D and higher levels of iPTH when compared to white patients^20^. In a US study with 139,328 patients including 32% African-Americans on thrice weekly hemodialysis treatment in a single large dialysis organization, most laboratory values were measured monthly for up to 60 months (July 2001 to June 2006). The study found that African-Americans had higher serum calcium and PTH levels but similar concentrations of phosphorus and alkaline phosphatase and were more likely to receive injectable active vitamin D medications and at higher doses than their non-African-American counterparts^21^.

*Diabetes Mellitus* is known to be associated with low-remodeling bone disorder^12^^,^ 16 ^,^ 22. In our study, 39.3% of the patients with PTHi levels below 150 pg/mL had *diabetes mellitus*, consistent with the recent review by Bover *et al*., in which low-remodeling bone disorder was associated, among other diseases, with *diabetes mellitus* 13. Diabetes patients with CKD have lower rates of bone formation, a complication that often precedes CKD development. In these patients, there is a deficiency of vitamin D and an increase of advanced glycated end-products (AGE) as well as a decrease in osteoblast lifespan^22^. Concerning therapy modality, several authors have found lower levels of PTHi in PD than in HD^23^^,^ 24. However, no difference was observed in our study, probably due to the low number of patients on PD.

High-remodeling bone disorder develops early during CKD and continues evolving when patients start dialytic therapy^25^. This was also observed in this study: a higher prevalence of serum iPTH levels was associated with longer therapy duration.

Regarding drug usage, we found a high percentage of patients using calcium-based phosphate binders (55.6%), consistent with the study by Tentori *et al.*, through DOPPS I, II, and III data analysis of 25,558 patients on hemodialysis, which showed a high percentage of these drugs (72.9%)^26^. Those authors also found that 17.1% used sevelamer compared with 14.7% found in our study. The 2013 Brazilian Chronic Dialysis Survey indicates a higher usage (38%) of non-calcium phosphate binders (Sevelamer)^4^, in disagreement with our study, probably due to problems of the public health care system related to drug distribution.

The usage of active vitamin D and analogues (oral and venous calcitriol, alfacalcidol, paricalcitol) in the population studied was 40.2%. However, when analyzing distinct iPTH groups, we observed that 46.7% of the patients with iPTH > 1000 pg/mL used these drugs, while in patients with iPTH < 150 pg/mL 30.7% used them. Because of the cross-sectional nature of this study, some patients might have had a decrease in iPTH associated with the usage of vitamin D or previous (before 2013) parathyroidectomy. Compared to other studies, the use of vitamin D in the present study was low. For instance, in the study by Tentori *et al.*, vitamin D was prescribed to 52% of the patients, although this was not analyzed as a function of iPTH level^26^. An Argentinian study revealed that 59.3% of the patients with iPTH > 300 pg/mL, and 36.9% of the patients with iPTH < 150 pg/mL used active vitamin D or its analogues^15^.

Taking into consideration the large number of patients with hyperparathyroidism in our study, calcimimetics (Cinacalcet) were underutilized, probably because, even though they are available in Brazil, these drugs are still not freely distributed through the public health care network, unlike calcitriol and other active vitamin D analogues (*e.g.*, alfacalcidol). Similarly, the low usage of selective vitamin D receptors activators might be due to same reason.

There was no record of aluminum-based binders in the population studied, and hemodialysis is performed through reverse osmosis in the 11 nephrology centers. Aluminum serum levels were associated with lower iPTH levels in our study. Several authors question whether distinct aluminum sources, such as aluminum household utensils, parenteral solutions or foods, could explain this fact^27^^-^ 29.

Concerning the dialysate calcium concentrate (DCC), a recent study by Jean and Chazot on hemodialysis shows that different regions of the world have various strategies in relation to this topic. Decreasing the DCC slightly reduces calcemia, but mainly stimulates parathyroid hormone secretion and bone turnover. Conversely, increasing the DCC increases calcemia slightly and reduces parathyroid hormone secretion and bone turnover markedly. Furthermore, higher DCCs favor hemodynamic stability and can prevent ventricular arrhythmias. Even though some studies have shown that using individualized DCCs of 1.25 or 1.75mmol/L is not harmful, the real benefits of this strategy need to be assessed in a large, multicenter trial^30^. In peritoneal dialysis, long-term (1-2 year) use of low-calcium dialysate (LCD) with 1.25 mmol/L calcium concentration results in decreased serum total and ionized calcium levels and has no effect on phosphate level. No clinical significance in the low calcium change of standard calcium (SCD) bone metabolism was observed between LCD and SCD patients despite low calcium the increase of serum parathyroid hormone in LCD group^31^.

Based on the 2011 Brazilian Census of Parathyroidectomy, 10.7% of the patients on dialysis presented an iPTH > 1000 pg/mL^9^, which is in agreement with the results found in our study (11.8%). There is no accurate data on the number of parathyroidectomies performed in Brazil. However, a study in Argentina, with a population sample of 1210 patients, showed that 70 patients (5.8%) had been submitted to this procedure^15^. As mentioned, no references to parathyroidectomies were found in the records analyzed in our study.

The cross-sectional design of this study is a limitation and therefore some inferences cannot be made. Ionic calcium was not measured and only one iPTH measurement was performed. The importance of the present study is that, first, it looks at the reality of the dialysis units that serve a large part of the population; and second, it provides a general idea of practice patterns in Brazil, since it does not focus exclusively on data from symptomatic patients or from patients followed up in a CKD-MBD reference center.

We can conclude that the prevalence of patients outside the KDIGO iPTH target was 50.5%. Hyperphosphatemia occurred in 35.8% of the studied population and hypophosphatemia in 13.5%. Analysis of the serum calcium showed that 39.3% of the patients had hypercalcemia while 12.6 % presented with hypocalcemia. There was low usage of active vitamin D, selective vitamin D receptors activators, and calcimimetic drugs. These data draw attention to the need for stronger compliance with public policies and guidelines regarding the supply of drugs associated with CKD-MBD.
